# A Bit-Tracking Knowledge-Based Query Tree for RFID Tag Identification in IoT Systems

**DOI:** 10.3390/s22093323

**Published:** 2022-04-26

**Authors:** Yuan-Cheng Lai, Shan-Yung Chen, Zelalem Legese Hailemariam, Chih-Chung Lin

**Affiliations:** 1 Department of Information Management, National Taiwan University of Science and Technology, Taipei 106, Taiwan; shann.yung@msa.hinet.net (S.-Y.C.); d10709801@mail.ntust.edu.tw (Z.L.H.); 2 Department of Computer Information and Network Engineering, Lunghwa University of Science and Technology, Taoyuan 333, Taiwan; chchlin@mail.lhu.edu.tw

**Keywords:** RFID, knowledge-based, query tree, anti-collision protocol, bit tracking

## Abstract

In an IoT (Internet of Things) system where each IoT device has one/many RFID tags, there might be many RFID tags. However, when multiple tags respond to the reader’s interrogation at the same time, their signals collide. Due to the collision, the reader must request the colliding tags to retransmit their IDs, resulting in higher communication overhead and longer identification time. Therefore, this paper presents a Bit-tracking Knowledge-based Query Tree (BKQT), which uses two techniques: *knowledge*, which stores all the tag IDs that can possibly occur, and *bit tracking*, which allows the reader to detect the locations of the collided bits in a collision slot. BKQT constructs a query tree for all possible tags, called a k-tree, by using knowledge while it constructs bit-collision cases and the corresponding actions for each node in this k-tree by using bit tracking. In the identification process, BKQT traverses this constructed k-tree and thus identifies the colliding tags faster by taking the actions according to the happening bit-collision cases. From the simulation results, BKQT can improve the identification time by 44.3%, 46.4%, and 25.1%, compared with the previous knowledge-based protocols, Knowledge Query Tree (KQT), Heuristic Query Tree (H-QT), Query Tree with Shortcutting and Couple Resolution (QTSC), respectively.

## 1. Introduction

Radio Frequency Identification (RFID) is a wireless communication technology that enables data gathering and identifies any tagged object. Due to its automatic identification capability through radio frequency communication, it has become very popular. RFID systems have been widely used in locating and tracking objects in warehouse management, goods inventory, IoT systems, and other aspects of daily lives. The main components in an RFID system are a reader and multiple tags. Each tag has a unique ID, and the reader interrogates tags through communication over a shared wireless channel.

When multiple tags reply to the reader simultaneously, their signals collide. Due to the collision, the reader must request the colliding tags to retransmit their IDs, resulting in higher communication overhead and longer identification time. Thus, an efficient anti-collision protocol is necessary, especially when many tags exist in a typical IoT system. The basic idea of all the protocols is to enable the acknowledging of tags or fewer collisions for faster identification. Various RFID anti-collision protocols have been developed, such as Aloha-based protocols [1,2,3,4,5,6,7,8] and Tree-based protocols [9,10,11,12,13,14,15]. Aloha-based protocols, such as Aloha, Slotted Aloha, and Frame Slotted Aloha, estimate the number of tags and assign the proper number of slots to reduce the probability of tag collisions. Tree-based protocols, such as Binary Tree protocols (BT) [9,10,11] and Query Tree protocols (QT) [12,13,14,15], continuously split colliding tags into subsets until there is only one tag in a subset to be identified successfully. BT uses a binary random number to split the colliding tags, while QT splits the colliding tags according to their IDs. Thus, the latter requires lower system requirements in tags than the former, but the latter has the problem that the distribution of tag IDs influences its performance.

Some RFID systems may have a database. Thus, the reader can know about the number of tags to be identified [16] or the tag IDs that can possibly occur; this is called “knowledge”. In some scenarios, the reader actually knows the IDs of the possible tags which might appear. For example, the RFID system in a supermarket often has a database containing the tag IDs of all the goods that are sold in the store. The RFID system in a manufactory often has a database containing the tag IDs of all the products made in its factory. However, as these possible tags might appear (and are called appearing tags) or might not appear, sometimes it is necessary to frequently identify them. For the example of the supermarket, each customer buys some goods (appearing tags), which should be identified for payment. An intuitive method is to query these possible tags with their IDs one by one. However, the identification efficiency is not good when the appearing tags only occupy a small portion of the possible tags. Currently, many studies on tag anti-collision exploit the knowledge. In some of these protocols, the reader repeatedly identifies the existing tags in the system; so, they have the knowledge of the tags that have been identified before and can identify whether these tags stay or leave [17,18,19,20,21,22,23]. On the other hand, some protocols have knowledge about all the tag IDs that can possibly occur [24,25]. Thus, if an RFID anti-collision protocol can utilize this knowledge, it should accelerate the tag identification.

Recently, some studies have proposed knowledge-based anti-collision algorithms, mainly including Knowledge Query Tree (KQT) [16], Heuristic Query Tree (H-QT) [24], and Query Tree with Shortcutting and Couple Resolution (QTSC) [25]. KQT separates tags from the known tag ID range into several intervals. The reader in KQT interrogates the tags in the same ID interval, and thus, it can limit the number of tags that respond simultaneously [16]. Moreover, H-QT creates a decision tree for the known tags and repeatedly selects the most efficient bit for subsequent query by traversing this decision tree [24]. Furthermore, QTSC constructs a knowledge-based query tree by utilizing the database and adopting shortcutting and couple-resolution techniques for the identification operation [25].

On the other hand, bit-tracking technology allows the reader to perceive the locations of collided bits in a collision slot; so, the bit-tracking anti-collision algorithms have attracted considerable attention recently due to their positive impact on decreasing the identification time [26,27,28,29,30]. A typical representative is the Collision Tree protocol (CT) [29,30]. In this protocol, a query tree using the bit-tracking technology can split the colliding tags from the position in the first collided bit. The theory proves that the performance of the CT is only dependent on the number of tags to be identified [30].

Using knowledge can help to split the colliding tags, while using bit tracking also can achieve the same objective. To the best of our knowledge, no previous study investigates the combination of using knowledge and bit tracking. However, the combination is not trivial because the splits determined by the knowledge-based tree and bit tracking might be in conflict. Thus, to obtain the best identification efficiency, we consider taking advantage of the combining of knowledge with bit tracking to present a bit-tracking knowledge-based query tree (BKQT) for the RFID tag identification. BKQT first constructs a query tree for all possible tags by using knowledge while it constructs bit-collision cases and the corresponding actions for each node in a query tree by using bit tracking. In identification, BKQT can traverse this constructed knowledge-based query tree and thus identify the colliding tags faster by taking the corresponding actions according to the happening bit-collision cases.

Our main contributions are summarized in the following:

- We first proposed the concept of combining knowledge and bit-tracking techniques.
- We developed a novel anti-collision protocol, BKQT, which is composed of four algorithms, K-Tree Construction, Bit-collision Cases, Action Preparation, and Identification Operation, which will be explained later.
- We evaluated the performance of BKQT compared with the related work, i.e., KQT, H-QT, and QTSC, by considering the number of total slots and the identification time subject to the number of appearing tags, the number of possible tags, tag ID length, and tag similarity.

The rest of this paper is organized as follows. Section 2 briefly describes the related work, including KQT, H-QT, and QTSC. Section 3 presents the concepts and operations and an example of our proposed protocol, BKQT. In Section 4, the simulation results of BKQT, compared with the related work, are exhibited. Finally, the conclusions are given in Section 5.

## 2. Related Work

### 2.1. Knowledge Query Tree (KQT)

KQT, which was modified from QT, takes advantage of knowing the number of possible tags identified and the range of their IDs [16]. It aims to solve the collisions more efficiently by limiting the number of tags responding simultaneously. In the beginning, the reader utilizes the known number of tags, *Nexp*, to divide the ID range into *Nexp* equiprobable intervals. Thus, the *i*-th interval, $I_{i}=[SP_{i}$, $EP_{i}$], where $SP_{i}$ and $EP_{i}$, respectively, represent the ID start point and endpoint in this interval, is likely to have only one tag. Then, the reader performs the identification process for the *Nexp* cycles, i.e., one cycle for each interval. In cycle *i*, the reader broadcasts $SP_{i}$ and $EP_{i}$. All tags having their IDs located in this interval reply to their IDs. If only one tag responds, the reader can identify it and starts the next cycle. When a collision occurs, the reader starts a general QT procedure. This process is performed repeatedly until all the intervals are completed. As the knowledge used in this protocol is based on a specific block of tag IDs, it has a better performance than QT when the tag IDs are concentrated in a particular zone.

### 2.2. Heuristic Query Tree (H-QT)

H-QT extends the query tree protocol to take advantage of the known tag IDs to identify the appearing tags [24]. H-QT protocol creates a decision tree with knowledge and repeatedly selects the most efficient bits for subsequent queries. The first key contribution of H-QT is *heuristic*: H-QT selects the most efficient bits heuristically by matching known tags as candidates and querying the tags dynamically. The second contribution is *practicality*: many known tags can be used as candidates for the appearing tags in the reader’s interrogation range in H-QT practicality. The third contribution is *efficiency*. H-QT protocol is scalable because it has the time complexity O(log *n*).

Practically, H-QT models the knowledge-based anti-collision as a data classification problem. It uses entropy to calculate a quantitative measurer for the worth of an attribute as a question and defines the test attribute in most small decision trees using the Iterative Dichotomiser 3 (ID3) algorithm. The ID3 algorithm uses a statistical property called information gain measure to select the most efficient bit at each step when the tree grows. The construction process of this heuristic query tree continues until each branch has only one tag. In the subsequent identification process, the reader also chooses the most efficient query bit (i.e., the bit with the largest entropy) to query the tags. The process is repeated in this way until all of the appearing tags have been identified.

### 2.3. Query Tree with Shortcutting and Couple Resolution (QTSC)

QTSC consists of a reader with a database containing the IDs of all possible tags [25]. The reader first runs the QTSC operation that observes all possible tag IDs and constructs a knowledge-based query tree, abbreviated as k-tree. The k-tree is a binary tree in which each leaf node corresponds to a possible tag and every internal node must have two children, to avoid unnecessary collision and idle slots in the identification process.

In the identification process, the reader utilizes the k-tree to prepare proper bit-string queries to identify all the appearing tags in the reader’s interrogation range. QTSC further applies shortcutting and couple-resolution techniques in a collision slot during the identification process. Using the shortcutting technique can skip redundant queries in the k-tree while using couple resolution, which transmits two IDs simultaneously within the same slot and can skip redundant queries if the number of possible tags is two. Thus, QTSC effectively reduces the identification time when compared with the KQT and H-QT protocols.

## 3. Bit-Tracking Knowledge-Based Query Tree

BKQT mainly utilizes knowledge and bit-tracking techniques. It constructs a k-tree for all possible tags by using knowledge while it constructs bit-collision cases and the corresponding actions for each node in this k-tree by using bit tracking. The main concept of BKQT involves observing the locations of collided bits to determine which possible tags appear. For a clear understanding, we first use an example to explain this concept. Assuming that there are five tags 10000, 01001, 00100, 00010, and 00001. If the reader gets the bit response as X0XXX, where X means a collided bit, BKQT can detect that tags 00001, 00010, 00100, and 10000 appear, while tag 01001 is absent. The reason is as follows. The first bit being “X” (For 1-bit string, we use “to denote the string to avoid the confusion. However, for the multi-bit string, we omit “because it is very clear.) indicates that tag 10000 appears. The second bit being “0” implies that tag 01001 is absent, the third bit being “X” implies that tag 00100 appears, and the fourth bit being “X” implies that tag 00010 appears. The last bit being “X” implies that tags 01001 and/or 00001 appear. However, tag 01001 is absent, observing from the second bit. Thus the reader can infer that tag 00001 appears.

BKQT is composed of four phases: *K*-*Tree Construction*, *Bit*-*collision Cases*, *Action Preparation*, and *Identification Operation*. The k-tree construction phase constructs the k-tree with the knowledge of all possible tags. The bit-collision cases phase generates all possible bit-collision cases with regard to the collided bits in each node of the k-tree. The action preparation phase generates proper actions, such as “SPLIT”, “READ”, “ABSENCE”, and “COUPLING”, which will be explained later, based on the bit-collision cases. Finally, in the identification process, the identification operation phase takes the corresponding actions according to the happening bit-collision cases.

### 3.1. Notations

**Figure 1:**
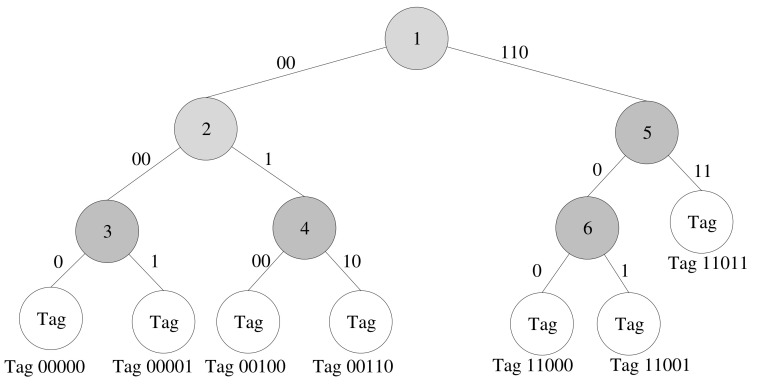
Example of k-tree. To be clearer, $S_{n}$ of a node *n* is labeled in the link connecting its parent. Thus, $S_{n}$ of the root node is ∅ (empty string).

The notations used in BKQT and their meanings are presented in Table 1. Regarding the knowledge, let *D* be the set of all possible tag IDs, and *d* is a possible tag ID, i.e., *d* ∈D. Regarding the k-tree construction phase, let T be a k-tree with its root as $T_{\pi }$. Each node n in the tree has two children, where $n_{l}$ and $n_{r}$ are its left and right children, respectively. Each node n has an associated substring, $S_{n}$, which denotes the bit string related to the query. Thus, the tag ID of each possible tag is formed by concatenating $S_{n}$ from the root node to its corresponding leaf node. Let $ID_{n}$ be the set of tag IDs under node n. For example, Figure 1 shows an example of a k-tree where node 2 has $S_{2}=00$ and $ID_{2}$ = {00000, 00001, 00100, 00110}.

Regarding the bit-collision cases phase, $Case_{n}$ is a set of bit-collision cases in node n. $Case_{n}$ is composed of combinations, $Z_{n},$ and bit response, $Y_{n}$, which will be explained later. Regarding the action preparation phase, notation a means an action, which might be “READ”, “ABSENCE”, “COUPLING”, and “SPLIT” for a bit-collision case. $Act_{n}$ is the set of a 3-tuple (*d*, $Y_{n,i}$, *a*), meaning that the action *a* for each tag ID d when bit response $Y_{n,i}$ happens in node *n*.

Regarding the identification operation phase, after the reader sends a query it will get a response, b, which is no signal or a bit string containing “0”, “1”, and “X”. Moreover, notations K and Q represent a stack of the queries used during the tag identification and a queue of the queries for the coupling tag IDs, respectively. In BKQT, F(), G(), and H() are the recursive functions used in k-tree construction, bit-collision cases, and action preparation, respectively.

### 3.2. K-Tree Construction

The k-tree construction phase constructs a k-tree with knowledge about all possible tags. The k-tree is a binary tree where each leaf node corresponds to a possible tag. Every internal node has two children to avoid unnecessary collision and idle slots on identification. The pseudo-code of k-tree construction is shown in Algorithm 1, where its input is a set of all possible tag IDs, and its output is a constructed k-tree. First, the tree is initialized as a root node $T_{\pi }$, which is a dummy node with $S_{T_{\pi }}$ as ∅ (empty string). For each tag, *d*, among all possible tag IDs, *D*, in the database, function F() will insert a new node for the tag by traversing from the root node (line 3).

Line 5–31 shows the pseudo-code of the function *F*(), which has three parameters: the beginning node to be traversed (*n*), the tag ID to be inserted (*d*), and the location to be compared (*i*). The idea of this function is to compare the prefix of *d* and $S_{n}$ of each node in traversing this tree. If they are the same, meaning the full match, BKQT should travel the k-tree deeper by visiting its left or right node by calling *F*() again. On the other hand, if they are different, meaning the partial match, BKQT should split this node and create one child for this new tag.

In line 6, *j* indicates the location to be compared in $S_{n}$ and is initialized as 1. Lines 8–14 shows what to do when the full match happens. When *j* is larger than the length of $S_{n}$, meaning the full match, we should travel the k-tree deeper. Thus, if $d\left[i\right]$ is equal to “0”, we continue to travel the k-tree from its left child by invoking $F\left(n_{l},b,i\right)$. In contrast, if $d\left[i\right]$ is equal to “1”, $F\left(n_{r},d,i\right)$ is invoked. On the other hand, lines 15–26 describe how to do splitting and create a new node if there is a partial match, i.e., $S_{n}$[j] is not equal to d[i]. In this case, the algorithm creates a new node *m* for splitting. If d[i] is equal to “0”, the algorithm creates a new node for this tag as the left child of *m* and sets the original node as its right child. In contrast, if d[i] is equal to “1”, the algorithm creates a new node for this tag as the right child of *m* and sets the original node as its left child. Finally, the algorithm sets the correct bit strings, $S_{m}$, $S_{m_{r}}$, $S_{m_{l}}$ for the changed nodes, m, $m_{r}$, and $m_{l}$.
**Algorithm 1.** Pseudo-code for k-tree construction**input**: *D* **output**: T 1. $T_{\pi }$ ← create a new node with $S_{T_{\pi }}=\emptyset$ 2. **for**
d∈*D* 3.   $T_{\pi }$ ← F($T_{\pi },d,\;1)$ 4. **end for** 5. **function**
$F\left(n,d,i\right)\;$ 6.   j ← 1 7.   **while**
*i* ≤ |d| **do** 8.    **if**
j > $|S_{n}|\;$**then**            //full match, so travel deeper// 9.      **if** $d\left[i\right]$ = “0” **then**          //travel left child// 10.      $n_{l}$ ← F ($n_{l}$, d, i) 11.     **else if**
$d\left[i\right]$ = “1” **then**     //travel right child// 12.      $n_{r}$ ← F ($n_{r}$, *d*,i) 13.     **end if** 14.     **break while** 15.    **else if**
$S_{n}\left[j\right]$ ≠ $d\left[i\right]$ **then**      //partial match, so do splitting // 16.     m ← a new node            //create a new node for splitting// 17.      **if**
$d\left[i\right]$ = “0” **then**           //create a left child// 18.      $m_{l}$ ← create a new node 19.      $m_{r}\;$ ← n 20.     **else if**
$d\left[i\right]="$1” **then**       //create a right child// 21.      $m_{r}\;$ ← create a new node 22.      $m_{l}$ ← n 23.     **end if** 24.     adjust $S_{m}$, $S_{m_{r}}$, $S_{m_{l}}$ for nodes *m*, $m_{r}$, and $m_{l}$ 25.     n ←m 26.     **break while** 27.    **end if** 28.     i←i+1; j←j+1 29.   **end while** 30.   **output**
n 31. **end function**

### 3.3. Bit-Collision Cases

The bit-collision cases phase generates all bit-collision cases in each node of the k-tree. However, in each node there are many combinations, where each combination means one instance of different possible tags appearing. Thus, in each node, we will generate many bit-collision cases, i.e., one bit-collision case for one combination. The pseudo-code of the bit-collision cases is shown in Algorithm 2. The input is the constructed k-tree. The output is a tree with all bit-collision cases in each internal node.

Lines 1–2 compute the tag ID collection, $ID_{n}$, under each node *n* in the tree T and create the bit-collision cases for each node by invoking function *G*() from the root node. Function $G\left(n\right)$ will recursively visit each node n to check whether the visited node is a leaf node or not. This recursively continues until it reaches the leaf nodes by invoking $G\left(n_{l}\right)$ and $G\left(n_{r}\right)$, as shown in lines 4–5.

The purpose of line 6 is mainly to limit the maximum number of combinations. When a node *n* has |$ID_{n}$| possible tags, the number of combinations is $2^{\left|n_{D}\right|}-1-\left|n_{D}\right|$, where 1 means the case that no tag appears, and $\left|n_{D}\right|$ means that no collision happens when only one tag appears. For example, in Figure 1, node 2 has $ID_{2}$ = {00000, 00001, 00100, 00110}; so, the number of its combinations is eleven, that is, combination 1 of node 2, $Z_{2,1}$ = {00000, 00001, 00100, 00110}, $Z_{2,2}$ = {00000, 00001, 00100}, $Z_{2,3}$ = {00000, 00001, 00110}, $Z_{2,4}$ = {00000, 00100, 00110}, $Z_{2,5}$ = {00001, 00100, 00110}, $Z_{2,6}$ = {00000, 00001}, $Z_{2,7}$ = {00000, 00100}, $Z_{2,8}$ = {00001, 00100}, $Z_{2,9}$ = {00000, 00110}, $Z_{2,10}$ = {00001, 00110}, and $Z_{2,11}$ = {00000, 00001}. However, to avoid having many combinations, which causes high computation overhead in generating bit-collision cases, we set a threshold, δ, to skip the nodes which will generate many combinations (line 6). For example, node 1, which has |$ID_{1}$| = 7, will be skipped if we set δ = 4. Afterwards, line 7 computes all combinations for a node. Notation $Z_{n}$ denotes the set of all combinations of node *n.*
**Algorithm 2.** Pseudo-code for bit-collision cases**input**: T **output**: T with the bit-collision cases in each internal node 1.  compute $ID_{n}$ in each node n ∈ T 2.  $G\left(T_{\pi }\right)$ 3.  **function** $G\left(n\right)$ 4.    **if**
$n_{l}$ ≠ leaf node **then**
$G\left(n_{l}\right)$ **end if** 5.    **if**
$n_{r}$ ≠ leaf node **then**
$G\left(n_{r}\right)$
**end if**
6.    **if** |$ID_{n}$| ≤ δ **then** 7.     $Z_{n}\leftarrow$ all combinations of *n* 8.     **for** (*i*=1; *i* ≤ |$Z_{n}$|, *i*++) **do** 9.      $Y_{n,i}=$ bit response of all tag IDs in $Z_{n,i}$ //the collided bit is marked as “X”// 10.         save a new case ($Z_{n,i}$, $Y_{n,i})$ into $Case_{n}$ 11.     **end for** 12.    **end if** 13. **end function**

For each combination, line 9 gets the bit response of all the tags in each combination. When the bits in an index of all the tags are the same, the response in this index should be the original bit; otherwise, the bit should be collided and marked as “X”. For example, for combination 4 in node 2, its bit response $Y_{2,4}$ = 00XX0 because $Z_{2,4}=\left\{00000,\;00100,\;00110\right\}$. Thus, we can create a new case, which is composed of a pair (combination, bit response), and save it into the set of bit-collision cases, $Case_{n}$ in node *n*.

### 3.4. Action Preparation

The action preparation phase generates the corresponding actions for each bit-collision case. From this bit collision, we can infer that some tags appear, some tags disappear, and some tags are uncertain. Thus, for the tag being judged to appear, the corresponding action is “READ”, meaning that this tag has been identified. For the tag being judged to disappear, the corresponding action is “ABSENCE”, meaning that this tag is absent. However, for the uncertain tag, the action is ‘COUPLING”, meaning that BKQT will execute coupling resolution to identify it. When the actions of all the tags in a node are “COUPLING”, meaning that no tag can be judged as appearing or disappearing, we use the action of “SPLIT”, representing that we traverse the k-tree deeper to speed up the identification. Algorithm 3 shows the pseudo-code of action preparation, where its input is the output of Algorithm 2, and its output is a k-tree with all the bit-collision cases and their corresponding actions in each internal node.

Action preparation will invoke the function H(), which visits each node recursively in the tree T and generates actions for each node. For node *n*, lines 3 and 4 check whether the left child $n_{l}$ and right child $n_{r}$ are the leaf nodes, respectively. If not, the function visits the node and constructs the actions for the left and right nodes recursively, which is similar to lines 4–5 in Algorithm 2.
**Algorithm 3.** Pseudo-code for action preparation**input**: *T* with the bit-collision cases in each internal node **output**: *T* with the bit-collision cases and their actions in each internal node 1.   $H\left(T_{\pi }\right)$ 2.   **function** $H\left(n\right)$ 3.    **if**
$n_{l}$ ≠ leaf node **then**$\;H\left(n_{l}\right)$ **end if** 4.    **if**
$n_{r}$ ≠ leaf node **then**
$H\left(n_{r}\right)$
**end if** 5.    **for** (*i*=1; *i* ≤ |$Z_{n}$|, *i*++) **do** 6.      D ← get the largest group of tag IDs in $Y_{n,i}$
7.     **for each** d∈D **do**
8.       **if**
d appears in all combinations, where combination *j* has $Y_{n,j}=Y_{n,i}$
**then**
a ← “READ” 9.       **else if**
d disappears in all combinations, where combination *j* has $Y_{n,j}=Y_{n,i}$ **then**
a ← “ABSENCE” 10.        **else** a← “COUPLING” **end if** 11.      **end if** 12.     add (*d,*
$Y_{n,i}$, *a*) into $Act_{n}$ 13.    **end for** 14.    **if** all actions in $Act_{n}$ are “COUPLING,” **then**
a ← “SPLIT” **end if** 15.   **end for** 16. **end function**

Lines 5–9 describe the procedure to find tag IDs that consistently appear (disappear) in all combinations which have the same bit response. For each bit-collision case, the algorithm first gets the largest group of tag IDs to make sure all possible tags will be checked. Then, the algorithm checks each tag in this group to find out whether it appears (disappears) in all combinations which will generate the same bit response. If yes, generating this bit response means that this tag ID appears (disappears). Thus, we can set the action for the tag as “READ” (“ABSENCE”). Otherwise, if a tag exists in some combinations and does not exist in other combinations, where these combinations generate the same bit response, we are not sure whether the tag appears or disappears; so, the algorithm sets the action “COUPLING” for this tag. Finally, line 12 adds the entry about the tag ID and the bit response and its action into an action table, $act_{n}$, for the node *n*. However, if no tag can be determined, i.e., the actions for all the tags are “COUPLING” in this node, setting the action as “SPLIT”, as the original QT does, will speed up the identification procedure.

### 3.5. Identification Operation

Algorithms 1–3 have a constructed k-tree, the bit-collision cases, and the corresponding actions in each node. For each identification process, BKQT executes the identification operation once. The basic concept of the BKQT identification operation phase is simple. It travels the k-tree and sends the proper query. When the reader gets the tags’ response, according to the bit-collision response and the currently visiting node, it matches this response with the bit-collision cases and adopts the corresponding actions from the matched bit-collision case. The reader arranges a stack, *K*, to store the queries in visiting the k-tree, and a queue, *Q*, to store the queues for couple resolution. Algorithm 4 describes the pseudo-code of the identification operation.

First, the reader puts the root node $T_{\pi }$ on the top of a stack *K* (line 1). Line 5 shows that if a node n in the tree is not a leaf node, the reader broadcasts its query, which is concatenated $S_{n}$, where *n* is from the root node to this node. The exception is that when the node is the root node $T_{\pi }$, the reader broadcasts “INITIALIZATION”.
**Algorithm 4.** Pseudo-code for identification operation**input**: T with the bit-collision cases and their actions in each internal node **output**: the identified tag IDs 1. K← $T_{\pi }$; Q←∅
2. **while**
K≠ empty **do** 3.   n ← pop the top node of K 4.    **if** (n is not leaf node) **then** 5.    broadcast concatenated $S_{n}$ **except if** n= $T_{\pi }$ **then** broadcast “INITIALIZATION” 6.    b ← tags’ response 7.    **switch**
*b* 8.      no signal: no tag                      //idle slot// 9.      no “X”: identify tag ID=*b*                //readable slot// 10.     having “X”:                           //collision slot// 11.       find (*d*, $Y_{n,i}$, *a*) in $Act_{n}$ where $Y_{n,i}=b$  //find a matched entry// 12.       **while** finding any new entry 13.        **switch** *a* 14.          “READ”: identify *d* 15.          “ABSENCE”: *d* disappears 16.          “COPUING”: insert d into Q 17.          “SPLIT”: push $n_{r}$ and $n_{l}$ into K; **break while** 18.        **end switch**                     //switch a// 19.       **end while** 20.    **end switch**                              //switch b// 21. **end while**

The reader gets the bit response *b*, which is no signal or a bit string containing bit “0”, bit “1”, and bit “X”. If the response has no signal, there is no tag appearing. If *b* does not have any “X” bit, the reader can identify the tag because only this tag appears. However, if b contains “X”, a collision happens. In this case, according to the actions corresponding to which bit-collision case matches *b*, the reader can identify some tags (READ), make sure that some tags disappear (ABSENCE), put some tags into the queue (COUPLING), or travel the k-tree deeper (SPLIT).

### 3.6. An Example of BKQT

Assume there are seven possible tags, which are 00000, 00001, 00100, 00110, 11000, 11001, and 11011. The reader starts constructing the k-tree for these seven possible tags following the k-tree construction algorithm (Algorithm 1). The example of a k-tree construction is presented as follows. The tree is initialized as a dummy node. The first tag, 00000, will be inserted into the left child of the tree’s root node. The algorithm then inserts the second tag 00001. When visiting node 00000, the algorithm meets a partial match and creates a new node, which has $S_{n}$ = 0000, for splitting. Then, it creates the right child for the new tag, which has $S_{n}$ = “1”, and uses the original node as its left child, which has $S_{n}$ = “0”. The algorithm will continue until all possible tags are inserted into the k-tree. The final constructed k-tree is presented in Figure 1. To be clearer, $S_{n}$ of a node *n* is labeled in the link connecting its parent. Thus, $S_{n}$ of the root node is ∅ (empty string).

Each node in the k-tree is visited during the bit-collision cases phase; so, the bit-collision cases in each node are generated. Before creating the bit-collision cases for node *n*, the algorithm (Algorithm 2) computes the set of possible tag IDs under node n*,* $ID_{n}$, and all its combinations of tag IDs,$\;Z_{n}$. For example, there are four possible tags in node 2; so, there are eleven $(2^{4}-1-4$) combinations. For the combination {00000, 00001}, the bit-collision response is 0000X. However, if the combination is {00000, 00100, 00110}, the bit-collision response is 00XX0.

Then, during the action preparation phase (Algorithm 3), it creates actions for each bit-collision case in each node. For example, if all tags under the root node respond to the reader, the reader gets the bit-collision response as XXXXX. Thus, the algorithm sets the action as “SPLIT” because no tag can be determined. Another case is when a bit-collision response in node 2 is 00XX0. First, we can infer that tag 00110 appears because the fourth bit is collided. However, as the fifth bit is “0”, tag 00001 is absent. As the third bit is collided and tag 00001 is absent, we can infer that tag 00000 appears. However, tag 00100 is uncertain because the result is still 00XX0 no matter if it appears or not. Thus, we can assign this action set as (00110, 00XX0, READ), (00000, 00XX0, READ), (00001, 00XX0, ABSENCE), and (00100, 00XX0, COUPLING).

During identification, assume the appearing tags are 00000, 00001, and 11000. The reader starts the identification process by sending an “INITIALIZATION” query. Then, all the appearing tags, which are 00000, 00001, and 11000, respond. The reader receives feedback with the bit-collision response XX00X (this bit-collision case cannot identify any tag so its corresponding action is “SPLIT”). Therefore, the reader travels the k-tree deeper, i.e., pushes 110 and 00 into the stack.

The reader continues to send query 00 popped from the stack in the next slot. Then the tags with prefix 00, which are 00000 and 00001, reply. However, a collision with the bit-collision response 0000X happens. In this case, the reader directly identifies both tags because they collide only at the last bit. Then, the reader sends query 110 popped from the stack and tag 11000 replies. This example of identification is shown in Table 2.

Table 3 shows another example, where the appearing tags are 00000, 00001, 00100, and 00110. The reader starts the identification process by sending the query “INITIALIZATION”. All appearing tags respond, so the reader receives feedback with the bit-collision response as 00XXX. In this case, the reader identifies two tags (00001 and 00110) and enqueues two tags (00000 and 00100) to the queue. In the next slot, the reader sends a coupling query, which includes 00000 and 00100, dequeued from the queue. The bit-collision response is 00X00; so, the reader can identify both tags (00000 and 00100).

## 4. Performance Evaluation

The performance evaluation of BKQT, compared to the previous knowledge-based protocols, KQT, H-QT, and QTSC, is exhibited in this section. We will investigate the effect of the number of appearing tags, the number of possible tags, the tag ID length, and the tag similarity, which means the length of the identical prefix in all tag IDs. Tag similarly was used in many papers [11,25,31] to investigate the locality of the tag IDs; so, we adopt the same definition as these papers. Each tag is assumed to own a 48-bit ID. Moreover, the tag IDs of all possible tags follow a uniform distribution, and the appearing tags are randomly selected from the set of possible tags. The simulations were implemented in Java programming language and were run on a computer with the CPU as Intel Core i7-6700 and the memory as 32.0 GB.

The evaluated performance metrics include the number of total slots and the identification time. A slot is defined as an identification period between a reader transmitting a query and the tags which match the prefix responding to their tag IDs. There are three kinds of slots: *collision*, *idle*, and *readable* slots. Collision slots are slots where multiple tags match the query; so, a collision occurs. Idle slots are the slots where no tag matches the query. Readable slots are slots where a single tag matches the query. Let $S_{C}$, $S_{I}$, and $S_{R}\;$ denote the number of collision slots, idle slots, and readable slots. Thus, the number of total slots, S, can be calculated as $S=S_{C}$ + $S_{I}$ + $S_{R}$.

The identification time is the time required to identify all appearing tags. Let $b_{C}$, $b_{I}$, and $b_{R}$ be the bit length of a collision slot, an idle slot, and a readable slot, respectively. Let T denote the identification time, and R denote the transmission bandwidth (bits/sec) of the RFID system. Thus, the identification time can be calculated as T = ($S_{C}$ × $b_{C}$ + $S_{I}$ × $b_{I}$ + $S_{R}$ × $b_{R}$)/R. The length of an idle slot can be significantly shorter than that of a collision slot and a readable slot. Thus, in our evaluation, $b_{I}$ is assumed as only a 3-bit length. Meanwhile, $b_{C}$ and $b_{R}$ are equal to the tag ID length, i.e., 48 bits. The RFID transmission bandwidth, *R*, is controlled at 640 kbps.

The default setting is that the number of appearing tags is 500, the number of possible tags is 1000, the tag ID length is 48 bits, and the tag similarity is 0. In the evaluation, we set δ=4 for BKQT. In all experiments, we only changed one parameter and kept the other parameters fixed as default values to easily observe the effect of the investigated parameter on the performance.

### 4.1. Appearing Tags

**Figure 2:**
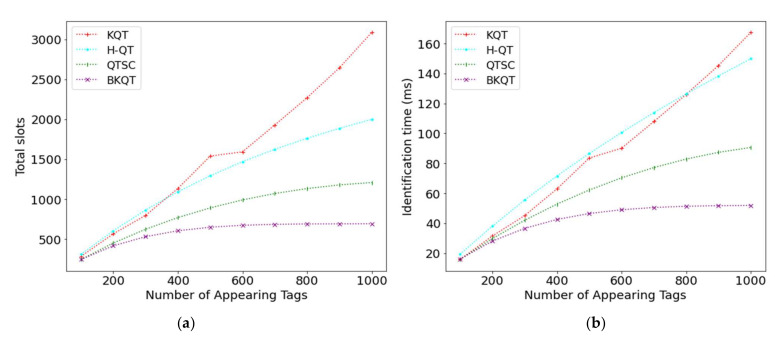
Total slots and identification time subject to the number of appearing tags. (**a**) Total slots, (**b**) Identification time.

Figure 2 shows the results of KQT, H-QT, QTSC, and BKQT by varying the number of appearing tags from 100 to 1000 tags. We can see that as the number of appearing tags increases, the number of total slots and the identification time increase. It is very reasonable that more appearing tags generate more slots and a longer identification time. From Figure 2a, KQT has the highest number of slots because it only uses the knowledge of the number of appearing tags, rather than the tag IDs. Its curve has a non-smooth increase in the number of tags between 500 and 600. This is because of the integer division of the number of tags in each interval, which is also shown in [16]. For example, when the tag ID range [000, 111] is divided into 3 intervals, the best division might be [000, 010], [011, 101], and [110, 111], where these intervals do not have the same number of possible tags. Compared with H-QT, QTSC has better performance because it uses the shortcutting technique to avoid some unnecessary collision slots and the couple-resolution technique to identify two tags at a slot in traversing the k-tree. Finally, BKQT has the least number of total slots because it not only uses the k-tree to inherit the advantages of QTSC but also uses the bit-tracking technique to further identify the colliding tags in a collision slot.

**Figure 3:**
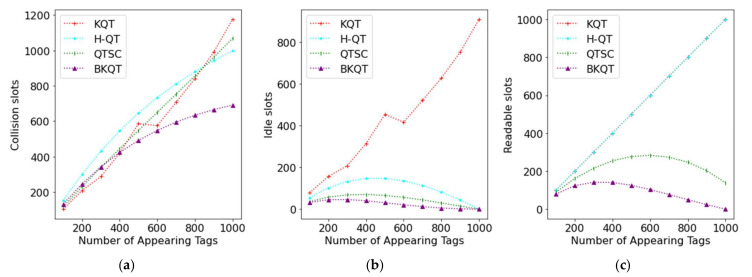
Collision, idle, and readable slots subject to the number of appearing tags (note that the curves of KQT and H-QT overlap in (**c**)). (**a**) Collision slots, (**b**) Idle slots, (**c**) Readable slots.

The tread on the identification time is very similar to that on the number of slots, except for the KQT curve. The number of total slots in KQT is obviously higher than that of H-QT, while they have a similar identification time. The reason is that KQT has many idle slots, which can be observed in the next figure (Figure 3b). In general, BKQT outperforms the previous work on the number of total slots and the identification time, no matter what number of appearing tags. Compared with KQT, H-QT, and QTSC, BKQT can improve 57.9% (1−648/1539), 49.8% (1−648/1292), and 27.2% (1−648/890), respectively, on the number of total slots and improve 44.3% (1−46.5/83.5), 46.4% (1−46.5/86.7), and 25.1% (1−46.5/62.1), respectively, on the identification time when the number of appearing tags is half of the number of possible tags, i.e., the number of appearing tags is 500.

The intermediate results shown in Figure 3 are exhibited to perceive that the number of collision, idle, and readable slots is subject to the number of appearing tags. Intuitively, the number of collision slots increases as the number of appearing tags increases. Figure 3a shows that KQT, H-QT, and QTSC are worse than BKQT. It is worth noting that KQT, H-QT, and QTSC have a similar number of collision slots. The reason is that when QTSC uses the couple-resolution technique, a collision slot can be regarded as identifying two tags. Thus, increasing the number of collision slots generated by the couple resolution will reduce the number of readable slots. We can see the significant reduction in readable slots achieved by QTSC in Figure 3c. However, BKQT still has the least number of collision slots. For the idle slots, KQT divides the ID range according to the number of possible tags; so, it will generate many idle slots, as shown in Figure 3b. From Figure 3c, KQT and H-QT have the same number of readable slots as they identify one tag in a readable slot. However, QTSC can use the couple-resolution technique to identify two tags in a collision slot, resulting in a lower number of readable slots. Finally, BKQT further uses the bit-tracking technique to identify many tags in a collision slot according to the locations of collided bits; so, it generates the least number of readable slots.

From Figure 3, BKQT outperforms other protocols on the number of collision slots, idle slots, and readable slots. Thus, BKQT definitely surpasses other protocols on the number of total slots and the identification time, as shown in Figure 2.

### 4.2. Possible Tags

**Figure 4:**
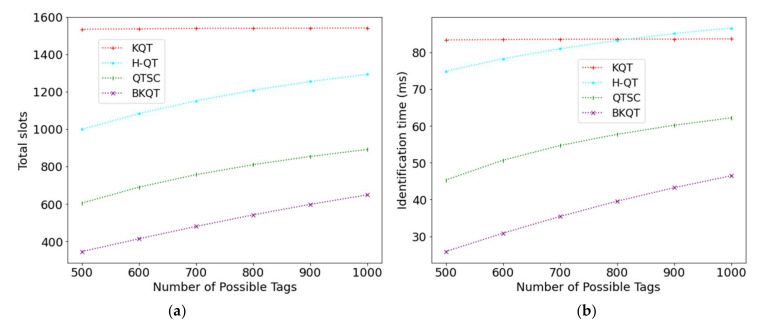
Total slots and identification time subject to the number of possible tags. (**a**) Total slots, (**b**) Identification time.

Figure 4 shows the results by varying the number of possible tags from 500 to 1000. When the number of possible tags increases, the number of total slots in KQT almost remains stable, while those of H-QT, QTSC, and BKQT slightly increase. Although KQT divides the tag ID range into many intervals, the number of possible tags will not change the tag ID range, resulting in a stable curve on the number of total slots. H-QT constructs a query tree, while QTSC and BKQT construct a k-tree. When the number of possible tags increases, the tree becomes larger and higher. Thus, in traversing the tree for querying, there is a higher possibility of collision and idle slots during the identification process. However, Figure 4a shows that BKQT reduces the number of total slots remarkably compared with the others, and their gaps are very obvious no matter what number of possible tags. For example, compared with KQT, H-QT, and QTSC, BKQT can improve 77.5% (1−345/1533), 65.4% (1−345/998), and 42.8% (1−345/603), respectively, when the number of possible tags is 500 and can improve 57.9% (1−648/1539), 49.8% (1−648/1292), and 27.2% (1−648/890), respectively, when it is 1000. The least number of total slots also causes the shortest identification time in BKQT, as shown in Figure 4b.

Note, too, that although H-QT outperforms KQT in terms of the number of total slots, their identification time is similar. This is because KQT will generate a lot of idle slots, which have a shorter length. The evidence is shown in Figure 3b.

### 4.3. Tag ID Length

**Figure 5:**
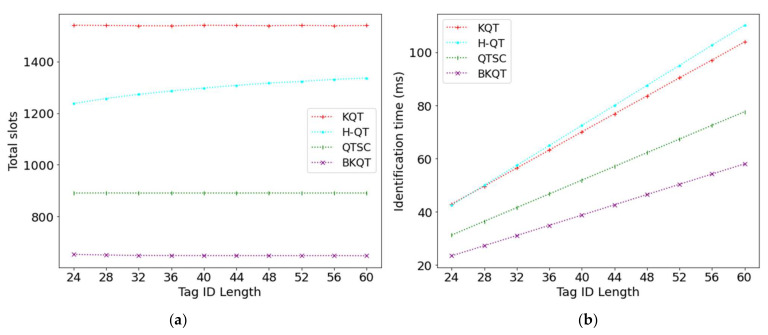
Total slots and identification time subject to tag ID length. (**a**) Total slots, (**b**) Identification time.

Figure 5 shows the results of four protocols by varying the tag ID length from 24 to 60. Figure 5a shows that the number of total slots in BKQT decreases while that of the other protocols remains stable. KQT divides the tag ID range into several intervals according to the known number of tags and tries to let each interval have only one tag. Thus, KQT is not affected by the tag ID length. On the other hand, H-QT and QTSC construct a query tree and a k-tree, respectively. As H-QT constructs a query tree, the longer tag ID length will cause a higher tree height, resulting in a larger number of total slots. However, in a k-tree, as each leaf node in the tree corresponds to a tag, the number of nodes in this tree and the tree’s height are fixed even when the tag ID length changes. Thus, QTSC keeps a stable number of total slots when the tag ID length varies. Although BKQT also constructs a k-tree, it further adopts the bit-tracking technique. When the tag ID length increases, the number of collided bits also increases; so, BKQT with the bit-tracking technique can identify more tags in a collision slot. Thus, BKQT can use fewer slots to identify all appearing tags.

On the other hand, the identification time of the four protocols increases. This is because enlarging the tag ID length will increase the length of the collision slots and readable slots. Thus, under a fixed number of total slots, the identification time almost linearly increases because most slots are collision slots and readable slots. In summary, Figure 5 shows that BKQT significantly outperforms other protocols on the total number of total slots and the identification time, no matter what the tag ID length.

### 4.4. Tag Similarity

**Figure 6:**
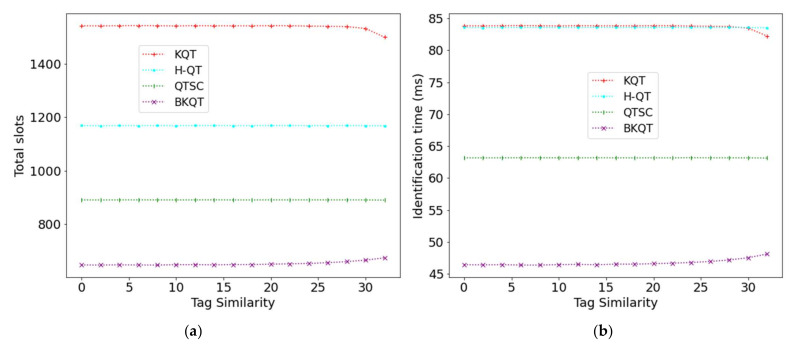
Total slots and identification time subject to tag similarity. (**a**) Total slots, (**b**) Identification time.

Figure 6 shows the results of four protocols by increasing the tag similarity from 0 to 32. When the tag similarity increases, the number of total slots of KQT, H-QT, and QTSC is almost kept fixed while that of BKQT increases. Although the tag ID range is reduced as the tag similarity increases, each interval divided by KQT is still expected to have one tag. Thus, KQT performance is not affected by tag similarity. On the other hand, tag similarity will not change the number of nodes in the tree and the tree’s height, causing H-QT and QTSC to both have a stable number of total slots. In contrast, when the tag similarity increases, generating fewer collided bits, the effects of using the bit-tracking technique in BKQT become a little bit smaller, resulting in more slots. More slots will cause a longer identification time, as shown in Figure 6b. However, although the performance of BKQT becomes a little worse when the tag ID similarity increases, it still considerably outperforms the other protocols.

## 5. Conclusions

The RFID technology has been widely used in various businesses, e.g., manufacturing, warehousing, transportation, retail outlets, IoT, and supply chains. Thus, to meet the different business requirements and provide better experiences, the tag collision problem should be conquered in order to quickly identify all the tags in the reader’s interrogation range. In this paper, a novel anti-collision protocol, BKQT, was proposed to efficiently solve this problem. BKQT first constructs a k-tree for all possible tags by using knowledge while it generates bit-collision cases and the corresponding actions for each node in this k-tree by using bit tracking. In identification, BKQT can traverse this constructed k-tree and thus identify the colliding tags faster by using shortcutting, couple resolution, and bit tracking, which can take the corresponding actions according to the happening bit-collision cases.

The simulation results show that BKQT significantly outperforms the previous protocols, KQT, H-QT, and QTSC, on the number of total slots and the identification time, no matter what number of appearing tags, what number of possible tags, what tag ID length, and what tag similarity. In the default setting, compared with KQT, H-QT, and QTSC, BKQT can improve 57.9%, 49.8%, and 27.2%, respectively, on the number of total slots and improve 44.3%, 46.4%, and 25.1%, respectively, on the identification time. From the intermediate results, we can also understand the BKQT can achieve the least number of collision slots, idle slots, and readable slots, to verify its outperformance.

## Figures and Tables

**Table 1:** Notations and their meanings used in BKQT.

**Notation**	**Meaning**
D	A set of possible tag IDs
d (d∈D)	A tag ID
T	A k-tree
$T_{\pi }$	The root node of the k-tree *T*
n, m	A node in the k-tree
$n_{l}$	The left child of node n
$n_{r}$	The right child of node n
$S_{n}$	A bit string corresponding to the query in node n
$ID_{n}$	A set of possible tag IDs under node n
$Case_{n}$	A set of bit-collision cases in node n
$Z_{n}$	A set of combinations of possible tag IDs in node $n.\;Z_{n}=\left\{Z_{n,1},\;Z_{n,2},\;\ldots ,\;Z_{n,\left|Z_{n}\right|}\right\}$, where $Z_{n,i}$ is the *i*-th combination
$Y_{n}$	A set of bit responses for $Z_{n}$. $Y_{n}=\left\{Y_{n,1},\;Y_{n,2},\;\ldots ,\;Y_{n,\left|Y_{n}\right|}\right\}$, where $Y_{n,i}$ is the bit response of the *i*-th combination
a	An action, either “READ”, “ABSENCE”, “COUPLING”, or “SPLIT”, for a bit-collision case
$Act_{n}$	A set of $\left(d,\;Y_{n,i},\;a\right)$, which stores the action a for each tag ID *d* when bit response $Y_{n,i}$ happens in node *n*
b	A response which is no signal or a bit string containing bit “0”, a bit “1”, and collided bit “X”
*K*	A stack to store the queries during identification
*Q*	A queue to store coupling tag IDs during identification
|*x*|	The length of *x*
**Function**	**Meaning**
F()	The recursive function used in k-tree construction
G()	The recursive function used in bit-collision cases
H()	The recursive function used in action preparation

**Table 2:** An example of BKQT identification.

Slot	Reader Query	Tags to Respond (00000, 00001, 11000)	Bit-Collision Response	Stack	Queue	Action
1	INITIALIZATION	0 0 0 0 0 0 0 0 0 1 1 1 0 0 0	XX00X (collision)	00, 110	∅	SPLIT
2	00	0 0 0 0 0 0 0 0 0 1	0000X (collision)	110	∅	(00000, READ), (00001, READ)
3	110	1 1 0 0 0	11000 (readable)	∅	∅	

**Table 3:** Another example of BKQT identification.

Slot	Reader Query	Tags to Respond (00000, 00001, 00100, 00110)	Bit-Collision Response	Stack	Queue	Action
1	INITIALIZATION	0 0 0 0 0 0 0 0 0 1 0 0 1 0 0 0 0 1 1 0	00XXX (collision)	∅	00000, 00100	(00001, READ), (00110 READ), (00000, COUPLING) (00100, COUPLING)
2	00000, 00100 (coupling)	00000 00100	00X00 (collision)	∅	∅	Identify both tags, 00000, 00100

## Data Availability

The data that support the findings of this study are available from the corresponding author upon reasonable request.
